# Impact of haemoglobin variants on the diagnostic sensitivity of glycated haemoglobin (HbA1c) assay methodologies in sub-Saharan Africa: a laboratory-based method validation study

**DOI:** 10.11604/pamj.2024.48.10.41679

**Published:** 2024-05-08

**Authors:** Priscilla Agatha Balungi, Anxious Jackson Niwaha, Rachel Nice, Lauren Rodgers, Nathan Mubiru, Rogers Mukasa, Angus Jones, Andrew Hattersley, Beverley Shields, Moffat Nyirenda, Timothy J McDonald

**Affiliations:** 1 Medical Research Council / Uganda Virus Research Institution &London School of Hygiene and Tropical Medicine, Uganda Unit, Entebbe, Uganda,; 2 Academic Department of Blood Sciences Laboratory, Royal Devon and Exeter NHS Foundation Trust, Royal Devon, United Kingdom,; 3 Institute of Biomedical and Clinical Science, College of Medicine and Health, University of Exeter Medical School, EX2 5DW, Exeter, United Kingdom

**Keywords:** Glycated haemoglobin (HbA1c), haemoglobin variants, HbA1c methodologies, continuous glucose monitoring

## Abstract

**Introduction:**

the utility of glycated haemoglobin (HbA1c) for the diagnosis and monitoring of diabetes in sub-Saharan Africa is uncertain due to limited data on the performance of the available HbA1c assay methods in this population, which has a high prevalence of haemoglobin variants. We aimed to compare the diagnostic accuracy of the major HbA1c methodologies (Boronate Affinity, Capillary Electrophoresis, High Performance Liquid Chromatography, Immunoassay) in an African population, and assess the impact of the common haemoglobin variant HbAS (sickle cell trait).

**Methods:**

whole blood samples were obtained from 182 individuals living with type 2 diabetes in Uganda. HbA1c values for each method were compared to average glucose measured over 14 days by continuous glucose monitoring (CGM). To determine concordance, the three HbA1c assay methods were compared to the capillary electrophoresis method.

**Results:**

there was a strong correlation between CGM average glucose levels and all four HbA1c methodologies (r=0.81-0.89) which did not differ in those with and without HbAS (present in 37/182 participants). The presence of HbAS did not alter the relationship between HbA1c and CGM glucose for any assay (p for interaction >0.2 for all methods). Diagnostic accuracy for CGM average glucose thresholds of 7 and 10mmol/L was similar across methods (area under the receiver operating characteristic curve 0.80-0.84 and 0.76-0.84 respectively). The maximum bias between the HbA1c assay methodologies was 2 mmol/mol (2.07%).

**Conclusion:**

all major HbA1c technologies offer accurate and comparable HbA1c measurement even in this population with high prevalence of haemoglobin variants.

## Introduction

Glycated haemoglobin (HbA1c) is the glycated portion of the haemoglobin compound formed through a non- enzymatic process during the life cycle of a red blood cell (~120 days). Stable HbA1c is proportional to the individual´s average glucose exposure over the preceding 90 days, which is clinically useful for the diagnosis and monitoring of diabetes mellitus [1]. HbA1c is measured by a number of methodologies, each exploiting differences in properties such as affinity, charge and immune-reactivity of the glycated haemoglobin molecule [2]. Changes in the amino acid sequence of the haemoglobin molecule caused by genetic mutations lead to presence of haemoglobin variants (for example amino acid substitution at position 6 (HbS), position 26 (HbE), and position 121 (HbD) [3,4]. The presence of these haemoglobin variants may interfere with the reliability of the HbA1c measurement [5]. The exact mechanisms leading to unreliable HbA1c measures in the presence of a haemoglobin variant are variable, but include alteration of glycation rates, red cell half-life or direct assay interference [6]. Modern HbA1c methodologies utilising chromatography or separation to quantify HbA1c are able to detect the presence of haemoglobin variants and validation calculators have been developed to correct for some of the more common variants [7,8]. Conversely, technologies that quantify HbA1c using the dye detection method (boronate affinity), or immuno reactivity method (immunoassay) do not offer information regarding the presence of the haemoglobin variants [5]. Up to 80% of the world haemoglobin variants are found in the sub-Saharan area of the African continent [9], but studies assessing performances of these HbA1c methodologies in this population are lacking. Therefore, it remains uncertain if the widely used HbA1c methodologies such as immunoassay, boronate affinity are reliable for diabetes diagnosis and monitoring within this population [10]. In this study, we aimed to assess the diagnostic sensitivity of the main HbA1c assays (Anion exchange High Performance Liquid Chromatography (HPLC), Capillary Electrophoresis, Immunoassay and Boronate Affinity) in the presence of haemoglobin variants, by comparison to the average glucose results calculated from Continuous Glucose Monitoring (CGM) device, and also to understand the degree of agreement between the three technologies (HPLC, immunoassay, boronate) in comparison to the capillary electrophoresis method which was used as the reference method.

## Methods

**Study setting and design**: this was a laboratory-based study which utilised samples from individuals participating in the Ugandan-based Optimal study. The optimal study was a cross sectional multicentre study that recruited type 2 diabetes patients from a rural based hospital (Masaka Regional Referral Hospital) and an urban based hospital (St. Francis Nsambya) who met the following inclusion criteria; diagnosed at the age of 18 years and above, more than 12 months´ diabetes duration, no initial insulin requirement for at least 1 year since the time of diagnosis, no change in glucose lowering therapy 3 months prior, and able to give informed consent [11]. The study received ethical review and approval from Uganda Virus Research Institution Review Board, Uganda National Council of Science and Technology (UNCST) and London School of Hygiene and Tropical Medicine ethics board (LEO).

**Laboratory testing**: blood was collected at clinic into Ethylene Diamine Tetra Acetic Acid (EDTA) blood collection tubes and then shipped to the testing laboratory within 6 hours at ambient temperature. All samples were tested same day on the immunoassay, capillary electrophoresis method before storage as whole blood at -80°C (for shipment to the United Kingdom for testing on the HPLC method).

**HbA1c measurement and detection of a haemoglobin variant**: HbA1c measurement was carried out at the Medical Research Council, Uganda Virus Research Institute & London School of Hygiene and Tropical Medicine (MRC/UVRI&LSHTM ) Uganda Research Unit, clinical diagnostics laboratory, on three different platforms: A) Capillary Electrophoresis on the Sebia flexi minicap piercing SN 94173 (Sebia technologies, Evry, France), manufacturer intra and inter assay precision; CV 1.2%; 1.5% respectively, B) Immunoassay method on Cobas 6000, c- model, SN 1493-16 (Hitachi high technologies corporation, Tokyo, Japan), manufacturer intra and inter assay precision; CV 1.1%, 1.5%, C) Boronate affinity method on the point of care instrument Afinion As100 analyzer 8; SN AS0024283 (Abbott rapid diagnostics technologies As, Oslo, Norway), manufacturer intra and inter assay precision; CV 1.2%,1.4%. In addition, samples were shipped to the Academic Department of Blood Sciences Laboratory, Royal Devon and Exeter NHS Foundation Trust, UK for a fourth methodology testing, on the Anion Exchange High Performance Liquid Chromatography method (HPLC) on the Tosoh G8 HPLC -723G8 (TOSOH, Tokyo, Japan), manufacturer intra and inter assay precision CV 0.4%, <2%, a method unavailable in Uganda. The choice of HbA1c methodology /instrument was based on; main detection method principle and also in country availability as shown in Table 1. All methods chosen for the study were standardised against the approved International Federation of Clinical Chemistry (IFCC) reference method for HbA1c measurement in human whole blood, which are traceable to DCCT/NGSP by calculation [12].

**Table 1:** summary of main HbA1c methodologies assessed in this study

Instrument	Methodology	Hb variant information	Advantages
Roche Hitachi cobas 6000	Immunoassay	No	High throughput. Not largely affected by variant AS, AD, AE, AC
Sebia flexi minicap piercing	Capillary electrophoresis	Yes	Information on presence variants Hb, Electrophoresis module on instrument
Afinion AS100 (point of care)	Boronate affinity	No	Ease of use, Quick turnaround time, Point of care
G8 Tosoh	Anion exchange high performance liquid chromatography	Yes	Information on HB fractions

**Stability testing**: in a sub study a set of whole blood samples (n=99) were tested for HbA1c before storage and after storage at -80 °C for one year to determine if the freeze thaw cycle would affect the stability of HbA1c analyte for samples that needed to be tested in a laboratory outside the country after a one-year storage.

**Continuous glucose monitor (CGM)**: continuous glucose monitoring was carried out using the Freestyle Libre Pro Flash Glucose Monitoring System (Abbott Laboratories, IIinois, USA) a professional continuous glucose monitoring device as previously described in the optimal study [13]. Interstitial glucose was recorded every 15 minutes for up to two weeks and raw glucose readings were downloaded from the Libreview software. Sensor data was considered for analysis if the total duration of CGM wear was at least 5 days. This test was used as the reference method since it is an independent marker of glycaemia which follows a different principle from the HbA1c methodologies.

**Figure 1:**
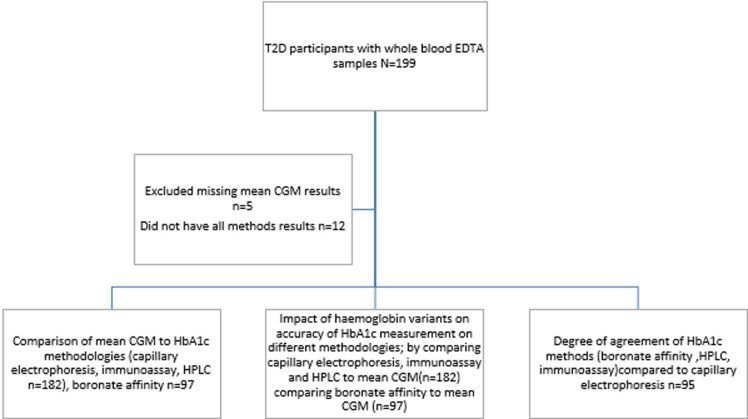
sample testing flow chart of studies on the impact of haemoglobin variants on accuracy of HbA1c results when compared to average glucose CGM results and diagnostic accuracy studies between the index methodologies being compared to a reference method

**Outcome data**: the average glucose data from the continuous glucose monitor (minimum of 5 days) was used as a comparator to the HbA1c results from the four instruments as shown in “Figure 1” flow chart. Out of 199 participants whose whole blood samples were available for Hba1c measurement,17 participants were excluded from analysis due to missing data (Figure 1). A total of 182 EDTA whole blood samples were available for HbA1c method comparison. Only 95 participants had HbA1c measurements assessed on the Point of Care Testing (POCT) from the Boronate Affinity method.

**Statistical analysis**: data was analysed using Stata V16.1 (stataCorp LLC, USA) software. Description of the participant baseline characteristics were expressed as mean (±SD) and proportions. We used scatterplots and Pearson correlation coefficients (r) to assess the association between HbA1c assay on each of the platforms to the average glucose readings from the Continuous Blood Glucose monitoring device. Lines of best fit for individuals with the variant and individuals without the variant were determined using linear regression analysis. To assess whether the slopes for those with the variant HbAS and those without were statistically different, an interaction term was used in the model (CGM glucose ~ HbA1c+ variant + (HbA1c*variant)). A two-tailed P<0.05 was considered statistically significant. We used the Bland Altman plots to examine the agreement between HbA1c results on three index methods i.e. Immunoassay, Boronate Affinity, HPLC as compared to the Capillary method which was selected as the reference method [14,15]. Acceptability of the HbA1c differences between the index and reference method were set at 3% (6.6%;48 mmol /mol) reference to total allowable limits according to NGSP [16,17] and at 6% which is the target value for CAP scheme for accuracy based studies (8.8%;73mmol/mol) [16].

## Results

Baseline participant characteristics are shown in Table 2. HbA1c values of included participants ranged from 24 to 183 mmol/mol, mean (SD); 72.1(±26.2) mmol/mol, aged 56 (±9.6) years. Sickle cell trait (HbAS) was present in 20.3% (37/182) of participants, and HbAE, HbAC was present in 0.5% (2/182) respectively.

**Table 2:** baseline characteristics for the study population (n=182, with the exception of Boronate Affinity HbA1c, available on 95 participants)

Description	Mean (±SD b), n(%)
Age-years	56 (±9.7)
Diabetes duration years mean	7.9(±6.2)
Female	125(58.7)
**Location**	
Rural	103(56.5)
Urban	79(43.4)
Mean Haemoglobin (g/dl)	14.1
**Haemoglobin type**	
HbA	143(78.5)
HbAS a	39(21.4)
**Individuals with anaemia (%)**	**16(8.8)**
HbA	12(8.4)
HbAS	4(10.3)
**Mean HbA1c by methodology (mmol/mol)**	
Boronate Affinity	70.9 (±23.2)
Anion high performance liquid chromatography	73.1(±27.3)
Immunoassay	72.8(±27.1)
Capillary Electrophoresis	71.4(±27.2)
Average glucose from CGM reader (mmol/L)	10.1 (±4.5)

aHbAS represents sickle cell trait, HbAC and HbAE, bSD represents standard deviation

**Effect of freeze storage on the performance of HbA1c results**: after one year of storage at -80 °C HbA1c increased modestly with a mean (SD) rise of 1.06 (±3.28) mmol/mol (P=0.0012) (percentage change 1.57%) on the immunoassay platform and decreased modestly when analysed by the capillary electrophoresis method with a reduction mean (SD) of -0.6 (±3.08) mmol/mol (p=0.08) with a percentage change -0.99%. Stability data was not collected on the Boronate Affinity method since the instrument manufacturer product details, state freezing EDTA samples as a contraindication for analysis on the platform.

**Figure 2:**
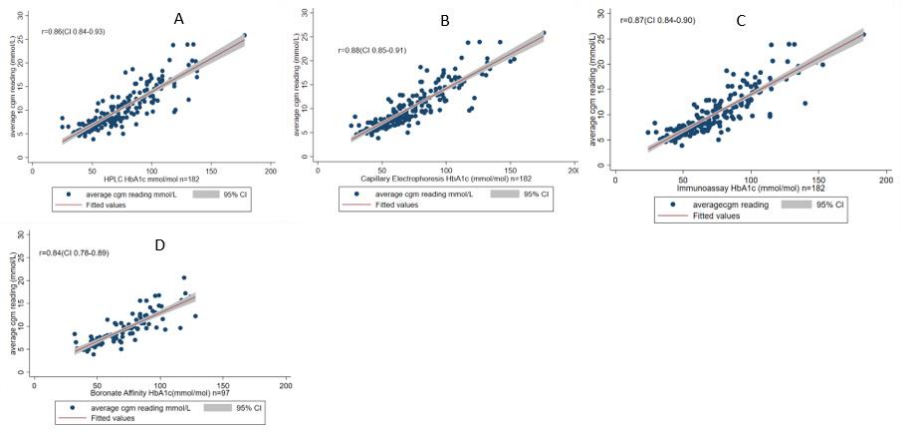
correlation graphs between the average CGM glucose results(mmol/L) and HbA1c(mmol/mol) of the different methodologies with a positive association represented by “R” and grey area representing 95% confidence interval for A) HPLC; B) capillary electrophoresis; C) Immunoassay; D) boronate affinity

**Correlation of average CGM glucose readings to HbA1c methodologies**: all four HbA1c methodologies showed a strong correlation with average CGM glucose readings for n=95 whole samples: HPLC r=0.86 (95% CI 0.84, 0.93); Capillary Electrophoresis r=0.88 (CI 0.85, 0.9); Immunoassay r=0.87 (CI 0.84,0.90); Boronate Affinity r=0.84(CI 0.78,0.89) as seen in Figure 2. The analytical accuracy for each HbA1c method in identifying participants with CGM average glucose of >7 and >10mmol/L was broadly similar across assays, with AUC ROC ranging from 0.80 to 0.84 for CGM glucose >7 mmol/L and 0.76 to 0.84 for CGM glucose >10 mmol/L (see supplementary data; Table 3).

**Table 3:** receiver operating curve results between average glucose results from CGM device (reader) and HbA1c methodology assay results at optimal and poor glycaemic control

HbA1c assay methodology	HbA	HbA	HbAS	HbAS	p-value
HPLCa	0.86(0.82,0.90)	19.2+ 5.65*CGM	0.87(0.76,0.93)	21.7+ 4.86*CGM	0.26
Capillary Electrophoresis	0.88(0.83,0.91)	15.0±5.63*CGM	0.89(0.79,0.94)	19.4±5.24*CGM	0.69
Immunoassay	0.86(0.82,0.90)	17.0 ± 5.59*CGM	0.89(0.79,0.94)	22.5+ 4.99*CGM	0.44
Boronate Affinity	0.84(0.77,0.90)	18.7+ 5.73*CGM	0.81(0.53,0.93)	22.6+ 5.39*CGM	0.37

aHPLC represents anion exchange high performance liquid chromatography HbA1c assay methodology

**Figure 3:**
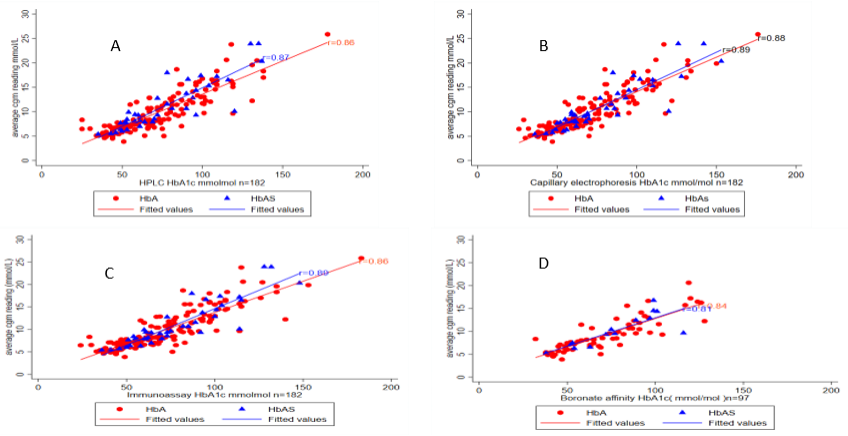
correlation graphs each having the Pearson correlation coefficients (R) value, between the average CGM glucose results(mmol/L) and different HbA1c methodologies (mmol/mol); A) HPLC, B) capillary electrophoresis, C) immunoassay, D) boronate affinity split by haemoglobin types “HbA and HbAS” whereby the circle represents the HbA type haemoglobin and triangle represents the HbAS

**Assessing the impact of a haemoglobin variant HbAS on the accuracy of HbA1c by assay methodology**: HbA1c methodology accuracy was not different in those with and without Sickle cell trait when compared to the average continuous glucose results, with no significant difference seen in intercept and slope for individuals with or without an HbS variant as shown in Table 4 and Figure 3.

**Table 4:** Pearson correlation coefficient (95%CI), linear regression equation interaction plots split by haemoglobin type (HbA vs HbAS) p value for each HbA1c assay method vs average glucose results from CGM device (reader)

Average glucose results CGM reader(mmol/L) (HbA1c)	HPLCa	Immunoassay	Boronate Affinity	Capillary Electrophoresis
> 7.0 mmol/L (53) mmol/mol	0.80(0.74,0.87)	0.83(0.77,0.89)	0.83(0.74,0.92)	0.84 (0.77,0.90)
> 10.0 mmol/L (63) mmol/mol	0.82(0.77,0.87)	0.82(0.77,0.87)	0.76(0.69,0.82)	0.84(0.80,0.89)

aHPLC represents anion exchange high performance liquid chromatography HbA1c assay methodology

**Figure 4:**
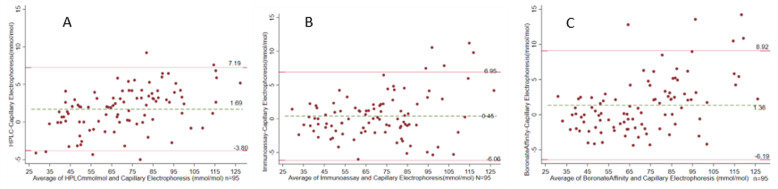
Bland Altman plots for A) HPLC B) immunoassay C) boronate affinity in comparison to capillary electrophoresis HbA1c method; the two straight lines represent the limits of agreement at 95%, and the dashed line represents the mean difference between the methods Supplementary data

**Concordance between HbA1c methodologies**: all methods showed strong concordance when compared to the Capillary electrophoresis reference method; as shown in Figure 4. The maximum percentage difference/bias between the index methods and reference method was 2.1% which is less than the defined NGSP acceptable limits of performance (+/-3% for HbA1c values; 48 mmol/mol ~ 6.6%).

**Figure 5:**
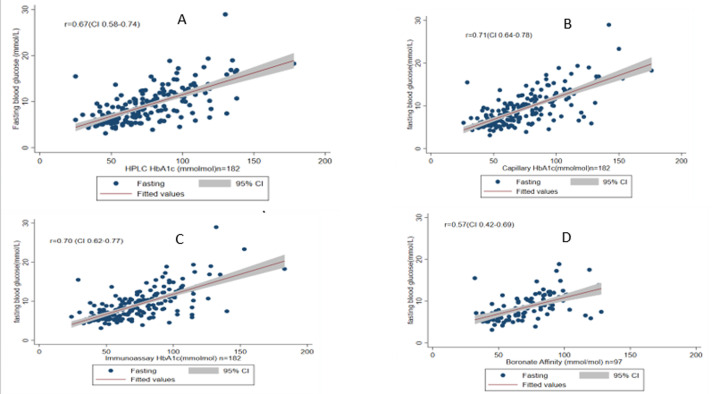
correlation graphs with a Pearson correlation coefficient (r) value at 95% confidence interval between the fasting blood glucose(mmol/L) and HbA1c methodologies(mmol/mol) A) HPLC; B) capillary electrophoresis; C) immunoassay; D) boronate affinity

## Discussion

In this study, all the major HbA1c methodologies we assessed showed good concordance with the HbA1c reference method and displayed a strong correlation to average CGM glucose values even in individuals with sickle cell trait. This data suggests that sickle cell trait, was the most common haemoglobin variant, and no impact on the diagnostic accuracy of these HbA1c methodologies was observed to assess glycaemia (as assessed by CGM). We observed minimal change in HbA1c concentration measured at baseline and after a one-year storage at -80°C on the capillary and immunoassay methods. These differences were within the manufacturer´s published batch to batch variations of these two methods (1.1-1.6% and 1.1-1.5% for capillary and immunoassay respectively). therefore, this difference is likely to be due to expected reagent batch to batch variation This implies that HbA1c can accurately be measured by capillary electrophoresis and immunoassay methods even after one year if the samples are appropriately stored at -80°C. Our study showed a good degree of agreement between HPLC, Immunoassay, Boronate Affinity HbA1c assay methods when compared to the capillary electrophoresis method among type 2 diabetes patients with a wide range of glycaemic burden (25-125 mmol/mol), with an average bias of < 2 mmol/mol (≤2.07%).

These results suggest that HbAS variant does not impact the accuracy of HbA1c assay methods and are in agreement with most studies performed in Caucasian populations as assessed by the HPLC, Capillary electrophoresis, immunoassay HbA1c assay methods[4,5], although the most common variants in this population are haemoglobin HbAS, C, D and E [1,8,19]. A study by Jaisson *et al*. did not observe any significant analytical interference from the presence of heterozygous C, S, E, D on the performance of HbA1c methodologies which use a separation technique (capillary electrophoresis, HPLC and affinity chromatography), when compared to a liquid chromatography mass spectrophotometry (LC-MS) reference method [20]. Similar findings were observed by Bouzid *et al*. in a study Tunisia, among individuals with sickle cell trait which demonstrated that sickle cell trait has no impact on the performance of HbA1c measured on a HPLC platform among individuals with type 2 diabetes which was a similar to our finding [20,21]. These findings were further confirmed in a similar experiment by Huang *et al*. HbA1c was strongly correlated to the average CGM even for individuals with sickle cell trait with a correlation of (r>0.80). In contrast to our study where we assessed the correlation of four major HbA1c assays including a point-of-care method, Huang *et al*. only compared HbA1c analysed with capillary electrophoresis to CGM [22].

In the current study, we have compared four HbA1c assays in the same study including a point-of-care method and more importantly against an independent measure of glycaemia. The major strength of our study in contrast to most previous studies is that we used CGM as an independent measure of glycaemic control burden to allow assessment of the relative performance of all the four HbA1c assay methods. A limitation of our study is that sickle cell trait was the predominant haemoglobin variant which was similarly observed in a study in Uganda [2,3] and our findings may not apply to sickle cell disease and other variants common in other parts of SSA. A further limitation in our study population was entirely those diagnosed with type 2 diabetes, with the majority having hyperglycaemia. It is possible that differences between assays may be more pronounced in those with normal glycaemia, and potentially impact utility of HbA1c for diabetes diagnosis for example a study in Tanzania by Kweka *et al*. showed that HbA1c underestimated the glycaemic burden for individuals with a sickle cell trait within the prediabetic range [24].

Our findings have general clinical implications especially for a region like Sub-Saharan Africa where haemoglobinopathies are common. For example the prevalence of sickle cell trait ranges from 10 to 40% of the sub-Saharan African population [9] with a high prevalence of sickle cell trait [2,5]. Although the Glycohaemoglobin Standardisation Program (NGSP) recommends that modern HbA1c immunoassays are not directly affected by the presence of haemoglobin variants like HbAS [26], further studies are needed to assess whether other haemoglobin variants such as HbC, HbF, HbE may affect the accuracy of HbA1c assays particularly those used in point-of-care devices. The good correlation and level of agreement displayed by the point of care device (using Boronate Affinity) versus the Capillary Electrophoresis method is reassuring and provides a pragmatic option to optimise glycaemic control assessment in a region where laboratory HbA1c accessibility is a challenge [2,7].

## Conclusion

Our findings suggest that the common HbA1c laboratory methods and the point-of-care boronate affinity assay are reliable even in the presence of sickle cell trait and can be used for glycaemic control assessment in sub-Saharan Africa.

### What is known about this topic

- *Presence of haemoglobin variants (which is highly common within the sub-Saharan populations) is likely to impact on the diagnostic accuracy of HbA1c. Previous studies within the East African region have shown limited access to laboratory HbA1c testing services and the performance of the HbA1c point of care devices in SSA is still limited*.

### What this study adds

- *The available laboratory HbA1c assay methods provide accurate HbA1c results for monitoring type 2 diabetes mellitus individuals and were not affected by the presence of heterozygous variant (HbAS);*
- *The HbA1c point-of-care method that utilises boronate affinity assay to measure HbA1c provides reliable results displaying a good agreement with the gold-standard laboratory methods and good correlation with average CGM glucose*.

## References

[1] Higgins T (2012). HbA1c--an analyte of increasing importance. Clinical biochemistry.

[2] Weykamp C (2013). HbA1c: a review of analytical and clinical aspects. Annals of laboratory medicine.

[3] Weykamp CW, Penders TJ, Muskiet FA, van der Slik W (2019). Influence of hemoglobin variants and derivatives on glycohemoglobin determinations, as investigated by 102 laboratories using 16 methods. Clinical Chemistry.

[4] Little RR, Roberts WL (2009). A review of variant hemoglobins interfering with hemoglobin A1c measurement. J Diabetes Sci Technol.

[5] Little RR, La´ulu SL, Hanson SE, Rohlfing CL, Schmidt RL (2015). Effects of 49 different rare Hb variants on HbA1c measurement in eight methods. Journal of diabetes science and technology.

[6] Thom CS, Dickson CF, Gell DA, Weiss MJ (2013). Hemoglobin variants: biochemical properties and clinical correlates. Cold Spring Harb Perspect Med.

[7] Marshall S, Barth J (2000). Standardization of HbA1c measurements: a consensus statement. Annals of clinical biochemistry.

[8] Schnedl WJ, Krause R, Halwachs-Baumann G, Trinker M, Lipp RW, Krejs GJ (2000). Evaluation of HbA1c determination methods in patients with hemoglobinopathies. Diabetes care.

[9] Jeremiah ZA (2006). Abnormal haemoglobin variants, ABO and Rh blood groups among student of African descent in Port Harcourt, Nigeria. African health sciences.

[10] Mongia SK, Little RR, Rohlfing CL, Hanson S, Roberts RF, Owen WE (2008). Effects of Hemoglobin C and S Traits on the Results of 14 Commercial Glycated Hemoglobin Assays. American Journal of Clinical Pathology.

[11] Niwaha AJ, Rodgers LR, Greiner R, Balungi PA, Mwebaze R, McDonald TJ (2021). HbA1c performs well in monitoring glucose control even in populations with high prevalence of medical conditions that may alter its reliability: the OPTIMAL observational multicenter study. BMJ Open Diabetes Research and Care.

[12] Hoelzel W, Weykamp C, Jeppsson J-O, Miedema K, Barr JR, Goodall I (2004). IFCC reference system for measurement of hemoglobin A1c in human blood and the national standardization schemes in the United States, Japan, and Sweden: a method-comparison study. Clinical chemistry.

[13] Niwaha AJ, Rodgers LR, Carr ALJ, Balungi PA, Mwebaze R, Hattersley AT (2022). Continuous glucose monitoring demonstrates low risk of clinically significant hypoglycemia associated with sulphonylurea treatment in an African type 2 diabetes population: results from the OPTIMAL observational multicenter study. BMJ Open Diabetes Res Care.

[14] Burtis CA, Ashwood ER, Bruns DE (2012). Tietz textbook of clinical chemistry and molecular diagnostics-e-book. Elsevier Health Sciences.

[15] Giavarina D (2015). Understanding bland altman analysis. Biochemia medica.

[16] Klonoff DC, Aron D, Cohen RM, Home P, John WG, Little RR (2019). The Need for Accuracy in Hemoglobin A1c Proficiency Testing: Why the Proposed CLIA Rule of 2019 Is a Step Backward. J Diabetes Sci Technol.

[17] Little RR, Rohlfing C, Sacks DB (2019). The National Glycohemoglobin Standardization Program: over 20 years of improving hemoglobin A1c measurement. Clinical chemistry.

[18] Almeida AM, Henthorn JS, Davies SC (2001). Neonatal screening for haemoglobinopathies: the results of a 10?year programme in an English Health Region. British journal of haematology.

[19] Gowda L, Vege S, Kessler D, Shaz B, Westhoff CM (2021). Screening of blood donors for sickle cell trait using a DNA-based approach: frequency in a multiethnic donor population. Transfusion.

[20] Jaisson S, Leroy N, Desroches C, Tonye-Libyh M, Guillard E, Gillery P (2013). Interference of the most frequent haemoglobin variants on quantification of HbA1c: comparison between the LC-MS (IFCC reference method) and three routinely used methods. Diabetes Metab.

[21] Bouzid K, Ahmed HB, Kalai E, Blibeche S, Couque N, Khiari K (2014). Prevalence of hemoglobin variants in a diabetic population at high risk of hemoglobinopathies and optimization of HbA1c monitoring by incorporating HPLC in the laboratory workup. Libyan Journal of Medicine.

[22] Huang J-H, Lin Y-K, Lee T-W, Liu H-W, Chien Y-M, Hsueh Y-C (2021). Correlation between short-and mid-term hemoglobin A1c and glycemic control determined by continuous glucose monitoring. Diabetology & Metabolic Syndrome.

[23] Ndeezi G, Kiyaga C, Hernandez AG, Munube D, Howard TA, Ssewanyana I (2016). Burden of sickle cell trait and disease in the Uganda Sickle Surveillance Study (US3): a cross-sectional study. The Lancet Global Health.

[24] Kweka B, Lyimo E, Jeremiah K, Filteau S, Rehman AM, Friis H (2020). Influence of hemoglobinopathies and glucose-6-phosphate dehydrogenase deficiency on diagnosis of diabetes by HbA1c among Tanzanian adults with and without HIV: A cross-sectional study. PLoS One.

[25] Serjeant G, Ndugwa C (2003). Sickle cell disease in Uganda: a time for action. East African medical journal.

[26] NGSP (2020). HbA1c methods: effects of Hemoglobin Variants (HbC, HbS, HbE and HbD traits) and Elevated Fetal Hemoglobin (HbF).

[27] Balde N, Camara A, Sobngwi-Tambekou J, Balti EV, Tchatchoua A, Fezeu L (2017). Improving access to HbA1c in sub-Saharan Africa (IA3) cohort: cohort profile. Pan Afr Med J.

